# The Effect of COVID-19 on Dental Education in Saudi Arabia

**DOI:** 10.7759/cureus.49721

**Published:** 2023-11-30

**Authors:** Lena S Elbadawi

**Affiliations:** 1 Periodontics, King Abdulaziz University, Jeddah, SAU

**Keywords:** questionnaire, outcome, perception, attitude, dental education, covid-19

## Abstract

Aim: The aim of this study was to comprehensively explore and evaluate the impact of the COVID-19 pandemic on dental education as perceived by dental students in Saudi Arabia.

Methods: Data were collected through a validated questionnaire from dental students at three universities. Demographic variables and students' experiences with online learning were examined. Statistical analysis involved one-way ANOVA and descriptive statistics.

Results: A total of 681 dental students participated, with a predominant male representation (76.8%). Statistically significant differences were found in the overall dental education experience, quality of education, and satisfaction with online dental education (p < 0.01). Group disparities were identified in specific aspects of online learning and concerns about losing clinical skills due to the pandemic lockdown. Variations were observed in satisfaction with compensatory measures for suspended clinics and perceived clinical skills acquired during COVID-19. The adoption of precautionary measures, such as N95 masks (36.1%) and face shields (42%), varied among students. Notably, only 36.9% reported seeing one patient per session.

Conclusion: This study reveals the many challenges faced by dental students during the COVID-19 pandemic in Saudi Arabia. While overall satisfaction was low, specific aspects like evening lectures received positive feedback. Concerns about clinical skills and varied responses to compensatory measures indicate the need for targeted interventions. Continuous monitoring of student experiences is crucial, and future research should delve into factors influencing satisfaction and long-term implications on clinical skills development.

## Introduction

The unprecedented global event of the COVID-19 pandemic has required swift and significant changes across many sectors, including education [1]. Saudi Arabia, much like the rest of the world, faced a sudden and critical need to transform its educational methods, particularly in fields that require hands-on training, such as dental education [2,3]. Prior to the pandemic, dental education in the Kingdom was characterized by in-person interactions, with students learning through direct patient care and mentorship in clinical settings. The compulsory shift to online learning platforms presented a unique set of challenges for both educators and students, who found themselves navigating uncharted territory with virtual classrooms and simulations replacing direct clinical experience [3,4].

This abrupt transition raised concerns about the efficacy of online dental education and the equity of access to these new learning modalities [5]. The change was not merely a technical one; it highlighted disparities in resources and access, with students from different backgrounds potentially facing significant barriers [3,5]. Educators had to rapidly adapt their teaching methods, often without prior experience or training in online education, which may have impacted the quality of instruction and students’ ability to learn effectively [1,3,5].

As dental education involves a high degree of practical skill acquisition, the implications of this enforced shift to online learning are particularly profound [6]. There is a question of how well virtual training can substitute for hands-on clinical experience, which is essential for developing the competencies required in the field of dentistry [7]. Furthermore, the psychological impact of the pandemic and the switch to remote learning on dental students' education, mental health, and future career paths remains to be fully understood.

Ensuring the continued production of skilled dental professionals in the face of unprecedented challenges is of paramount importance. To achieve this, studies should focus on informing future educational strategies by examining the perspectives of students who have experienced such obstacles. Therefore, the aim of this study is to comprehensively explore and evaluate the impact of the COVID-19 pandemic on dental education as perceived by dental students in Saudi Arabia.

## Materials and methods

The research protocol for this cross-sectional study received approval from the Research Ethics Committee at King Abdulaziz University Faculty of Dentistry (REC-KAUFD) with approval number 212-01-21, dated March 18, 2021.

Data collection was carried out using a questionnaire designed to assess the impact of the COVID-19 pandemic on dental education from the perspective of students. The questionnaire was disseminated digitally by sharing its link with the students. Prior to data collection, the questionnaire underwent a thorough content validity assessment by a panel of associate professors to ensure its appropriateness and relevance to the research objectives.

The study included dental students who met the following inclusion criteria: enrollment in King Abdulaziz University, Ibn Sina National College, or Taibah University and enrollment during the academic year 2019/2020, which marked the onset of the COVID-19 pandemic or before that year.

Prior to participating in the study, respondents were informed about the anonymity of their responses and their right to withdraw from the study at any stage without repercussions. Their digital consent was obtained before proceeding with the questionnaire.

The questionnaire was structured into two main sections. The first section collected information on respondents' gender, age group, city of residence, dental school, and current academic year. These demographic variables were essential for characterizing the study population. The second section of the questionnaire focused on gathering information regarding students' experiences and feedback on e-learning during the COVID-19 pandemic, which was the primary research focus.

The collected data underwent several steps of processing and analysis. Data cleaning was performed to identify and rectify any inconsistencies or errors in the collected data to ensure data quality. Data coding was then conducted to transform responses into a format suitable for quantitative analysis. For data analysis, we employed the Statistical Package for Social Sciences (SPSS), version 23.0, developed by IBM Corp., Armonk, NY. Descriptive statistics were used to tabulate demographic information and response frequencies and percentages. Bar charts were used to present percentages of questions with multiple items, particularly “How did your precautionary measures change in the clinic after the COVID-19 pandemic” and “What was the most common technical issue you encountered during online lectures, webinars, and studies during the COVID-19 pandemic?”. To explore significant variations in responses to survey questions, one-way ANOVA tests were conducted, with a significance level of p < 0.05 established to determine statistical significance.

## Results

In this study, a total of 681 dental students participated, with a predominant male representation (n = 523, 76.8%). Most respondents were in their sixth year (final dental school undergraduate year in Saudi Arabia) during the COVID-19 lockdown (n = 427, 62.7%). Further demographic details are provided in Table 1.

**Table 1:** Demographics of the participants (N = 681 students)

Variables		Frequency	Percentage
Gender	Male	523	76.8
	Female	158	23.2
Region of residence	Al Madinah	32	4.7
	Makkah	646	94.9
	Riyadh	3	0.4
City of residence	Jeddah	644	94.6
	Makkah	2	0.3
	Madinah	32	4.7
	Riyadh	3	0.4
Dental school	Ibn Sina National College	90	13.2
	King Abdulaziz University	559	82.1
	Taibah University	32	4.7
Academic year during the lockdown	2^nd^ (first dental school year)	8	1.2
	3^rd^	37	5.4
	4^th^	85	12.5
	5^th^	124	18.2
	6^th^ (last dental school year)	427	62.7

One-way ANOVA was employed to assess variations in survey responses among different groups. The findings revealed statistically significant differences in several key aspects of the participants' experiences during the pandemic. There was a significant divergence in the overall dental education experience (F(2, ∞) = 9.369, p < 0.01). Additionally, variations were observed in the quality of education (F(2, ∞) = 39.715, p < 0.01) and satisfaction with online dental education performance (F(2, ∞) = 88.361, p < 0.01) among the study groups.

Furthermore, the study identified significant differences in satisfaction levels with specific aspects of online learning. Understanding online information when lectures shifted to the evening exhibited group disparities (F(2, ∞) = 23.977, p < 0.01) as did ratings of trouble during the online study (F(2, ∞) = 16.502, p < 0.01). The examination of the online exam mechanism revealed substantial group variations (F(2, ∞) = 37.739, p < 0.05), encompassing factors such as time allocation, question quantity, image clarity, and technical issues.

The study also uncovered significant disparities in concerns about losing clinical skills due to the pandemic lockdown (F(2, ∞) = 150.152, p < 0.05). The impact of reduced clinic hours and precautionary measures on clinic performance showed significant variations among groups (F(2, ∞) = 25.18, p < 0.05). Additionally, satisfaction with compensatory measures for suspended clinics (F(2, ∞) = 58.469, p < 0.05) and clinical skills acquired after reduced requirements during COVID-19 (F(2, ∞) = 33.567, p < 0.05) differed significantly.

**Figure 1:**
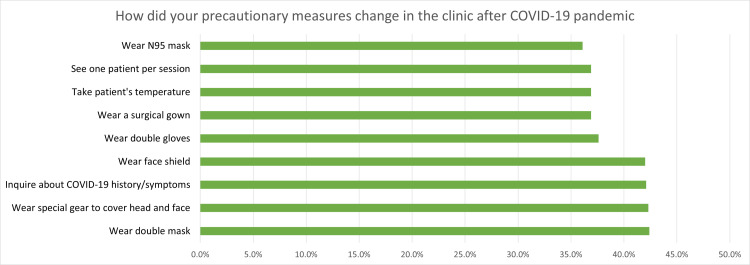
Answers to the question “How did your precautionary measures change in the clinic after the COVID-19 pandemic?” (N = 681 students)

**Figure 2:**
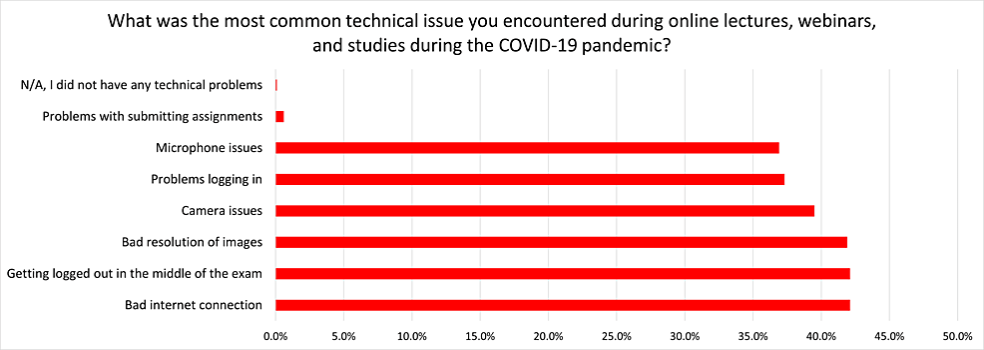
Answers to the question “What was the most common technical issue you encountered during online lectures, webinars, and studies during the COVID-19 pandemic?” (N = 681 students)

The likelihood of contracting COVID-19 demonstrated group variations (F(2, ∞) = 3.635, p = 0.027) as did the challenges of patient acceptance post-quarantine (F(2, ∞) = 11.941, p < 0.05). The quality of precautionary measures in clinics varied significantly among groups (F(2, ∞) = 25.391, p < 0.05). Lastly, comfort levels in entering clinics and treating patients post-COVID-19 vaccine administration exhibited significant group differences (F(2, ∞) = 108.569, p < 0.05). Descriptive statistics of students' experiences during and after the COVID-19 lockdown, precautionary measures in the clinic after the pandemic, and technical issues encountered during online lectures, webinars, and studies are presented in Table 2 and Figures 1, 2, respectively.

**Table 2:** Answers to questions regarding students’ experience (N = 681 students)

Question	Response	Frequency	Percentage
How satisfied were you with your overall dental education experience, considering the impact of the COVID-19 pandemic?	Very poor	145	21.3
	Poor	152	22.3
	Acceptable	215	31.6
	Good	110	16.2
	Very good	59	8.7
How satisfied were you with the quality of the education you received during the COVID-19 pandemic?	Very poor	72	10.6
	Poor	190	27.9
	Acceptable	226	33.2
	Good	170	25
	Very good	23	3.4
How satisfied were you with online dental education during the COVID-19 pandemic?	Very poor	86	12.6
	Poor	184	27
	Acceptable	238	34.9
	Good	114	16.7
	Very good	59	8.7
How satisfied were you with the evening lectures during the COVID-19 pandemic?	Very poor	52	7.6
	Poor	173	25.4
	Acceptable	219	32.2
	Good	176	25.8
	Very good	61	9
How satisfied were you with the experience of online exams conducted during the COVID-19 pandemic?	Very poor	150	22
	Poor	132	19.4
	Acceptable	202	29.7
	Good	114	16.7
	Very good	83	12.2
How satisfied were you regarding the technical challenges you faced during online studying, such as internet connection, laptop, and microphone issues?	Very poor	116	17
	Poor	149	21.9
	Acceptable	205	30.1
	Good	176	25.8
	Very good	35	5.1
How satisfied were you with the suspension of clinics for safety reasons during the COVID-19 pandemic?	Very dissatisfied	119	17.5
	Dissatisfied	174	25.6
	Neutral	196	28.8
	Satisfied	105	15.4
	Very satisfied	87	12.8
How satisfied were you with the implementation of the lockdown, given your concerns about the potential loss of your clinical skills and insufficient clinical training?	Very poor	161	23.6
	Poor	68	10
	Acceptable	216	31.7
	Good	149	21.9
	Very good	87	12.8
How satisfied were you with the impact of reducing clinics and clinical hours on your clinic performance?	Very poor	126	18.5
	Poor	132	19.4
	Acceptable	227	33.3
	Good	104	15.3
	Very good	92	13.5
How satisfied were you with the compensatory measures for clinics that were suspended during the lockdown, such as reducing requirements, the number of clinics, and the number of patients after reopening?	Very poor	95	14
	Poor	145	21.3
	Acceptable	229	33.6
	Good	179	26.3
	Very good	33	4.8
How satisfied were you with the level of clinical skills you acquired after the reduction in clinical requirements for some of your subjects during COVID-19?	Very poor	66	9.7
	Poor	139	20.4
	Acceptable	305	44.8
	Good	99	14.5
	Very good	72	10.6
How effective were the precautionary measures you implemented to prevent COVID-19 infection while working in the clinic with patients?	Very poor	88	12.9
	Poor	95	14
	Acceptable	242	35.5
	Good	148	21.7
	Very good	108	15.9
How comfortable do you feel entering clinics and treating patients now, given the administration of COVID-19 vaccines, increased public awareness and caution, and compliance with mandatory precautionary measures?	Very poor	79	11.6
	Poor	123	18.1
	Acceptable	170	25
	Good	134	19.7
	Very good	175	25.7
Did you get COVID-19?	Yes, after resuming clinics	92	13.5
	Yes, during lockdown	100	14.7
	No	489	71.8
Did you experience difficulties with patients being unwilling to come to your clinic after the quarantine period?	Yes	409	60.1
	No	272	39.9

## Discussion

The aim of this study was to comprehensively explore and evaluate the impact of the COVID-19 pandemic on dental education as perceived by dental students in Saudi Arabia. Our findings reveal a multifaceted and nuanced landscape of challenges and variations experienced by dental students during this unprecedented global event. In examining the overall dental education experience, significant differences among student groups emerged, shedding light on the diverse ways in which the pandemic has influenced the learning environment. The quality of education during this period also exhibited substantial variations, emphasizing the need for targeted strategies to address specific concerns. Our study further identified pronounced disparities in satisfaction levels with online dental education, pinpointing areas such as evening lectures and technical issues where tailored interventions may prove beneficial.

While a majority of respondents (54.1%) rated their overall dental education experience during the pandemic as "acceptable" or better, a significant proportion (43.6%) expressed dissatisfaction ("very poor" or "poor"). This indicates the considerable impact of the pandemic on the perceived educational quality. When assessing the quality of education received during the pandemic, only 28.4% of students rated it as "good" or "very good," suggesting that the abrupt transition to online learning and the suspension of clinical activities adversely affected educational quality.

The transition to online education received mixed responses, with 34.9% finding it "acceptable" and 39.6% rating it poorly. This reflects the challenges in replicating hands-on clinical training through online platforms. Notably, our findings regarding dissatisfaction rates are higher than those reported in previous studies conducted in Saudi Arabia and Oman, such as those by Al Rawahi et al. and Bahanan et al. [8,9], but align closely with the results reported by Abdul et al. [10]. This discrepancy could be attributed to differences in the timing of data collection. In the studies by Al Rawahi et al. and Bahanan et al., responses were collected during the lockdown period, early in the pandemic, while our study and Abdul et al.'s study collected responses later after the lockdown had concluded and students had returned to traditional learning. This temporal difference introduces the possibility of recall bias, and the responses of students who did not perceive online learning negatively might have been influenced or masked by the psychological stress and trauma inflicted by the pandemic. Studies have indicated a significant impact of the pandemic on the mental health of dental students [11,12], which could have influenced their perceptions and responses in our study and that of Abdul et al.'s study.

Evening lectures garnered widespread approval, with 58.8% rating them "acceptable" to "very good." In contrast, online exams received a less favorable response, with only 29.9% reporting a positive experience. Interestingly, our findings align with Mohsin et al.'s study involving students from various Saudi universities [6] but differ from Moothedath's study in Kerala, India, where over 60% of students viewed online exams positively [13]. It is worth noting that our sample is primarily from King Abdulaziz University in Jeddah, while Mohsin's participants are mainly from King Saud University in Riyadh. This similarity in findings could suggest a shared approach to dental education in Saudi Arabia, likely influenced by the centralized regulation by the Ministry of Health during the pandemic [14,15].

On the contrary, Moothedath's results may highlight global variations in how different countries approached online learning during the pandemic. For instance, while less than 30% of Moothedath's sample encountered technical issues, over half of our participants experienced at least two different forms of them, with barely 30% reporting their online experience to be a smooth one [13]. Additionally, Mohsen's study participants predominantly agreed that their evaluation and examination procedures were either postponed or canceled, emphasizing the diverse strategies employed by educational institutions worldwide [6].

Opinions were divided on the suspension of clinics, possibly reflecting concerns about the trade-off between hands-on experience and safety measures. Interestingly, 45.5% expressed worry about potential losses in clinical skills due to lockdown and reduced exposure. Post-lockdown, clinic performance was considered "acceptable" or better by 62.1%, indicating resilience in adapting to new clinical learning environments. Compensatory measures had mixed reviews, with 33.6% finding them "Acceptable" and 35.3% unsatisfied, a challenge in striking a balance between education and safety. Despite reduced clinical requirements, a robust 70.1% felt their clinical skills were at an "acceptable" level or better. These findings somewhat align with Kumar et al.'s report where less than half of interns agreed that the pandemic affected their confidence and readiness for clinical practice [16]. Similarly, they echo Javed et al.'s findings where most undergraduate students demonstrated moderate to good confidence and attitude toward their clinical competency and achieving all clinical requirements post-pandemic [17].

The adoption of precautionary measures by dental students during the COVID-19 pandemic exhibited variations. Notably, a minority (36.1%) reported wearing N95 masks, indicating a limited uptake of this high-level protective gear, while face shields were used by a comparable percentage (42%). In contrast, the utilization of surgical gowns was reported by 36.9%, suggesting a less widespread embrace of this measure. Temperature checks on patients were conducted by 36.9%, emphasizing a recognized but not universally applied precautionary practice. Inquiring about patients’ COVID-19 history or symptoms was reported by 42.1%, reflecting a moderate level of vigilance. Intriguingly, only 36.9% reported seeing one patient per session, implying that a majority adhered to a more traditional patient flow despite the challenges posed by the pandemic. It is noteworthy that these findings diverge somewhat from previous figures reported by Almarghlani et al. at the same university (King Abdulaziz University), where face shields were used by 78.3%, N95 masks by 61.3%, surgical gowns by 73%, and temperature checks by 91.7% [2]. This disparity could be attributed to the timing of data collection as Almarghlani et al.'s study was conducted during the peak of the pandemic, while our data were collected later [2]. This temporal difference introduces the possibility of recall bias, and it is plausible that participants in our study based their responses on their current situation rather than their circumstances during the lockdown.

Several limitations must be acknowledged in interpreting the results of this study. First, the retrospective nature of our survey poses a risk of recall bias as participants may have difficulty in accurately recalling their experiences during the early stages of the pandemic. Additionally, the timing of data collection, occurring after the lockdown, might have influenced responses, potentially masking the immediate impact of online learning. The generalizability of our findings may be constrained by the focus on a specific institution (King Abdulaziz University), and caution should be exercised when applying these results to other dental education settings. Moreover, there is a certain homogeneity in the demographics of our sample, which limited our ability to statistically test the potential effects of different factors related to demographics or backgrounds on the reported experiences. This restriction hinders the exploration of potential nuances within the student population. Furthermore, the variability in pandemic response measures across different regions and countries might limit the global generalizability of our findings. Lastly, the self-report nature of the survey may introduce social desirability bias as participants may provide responses they perceive as more socially acceptable.

## Conclusions

This study aimed to comprehensively explore the impact of the COVID-19 pandemic on dental education, focusing on the perceptions of dental students in Saudi Arabia. The findings reveal a complex landscape of challenges and variations, emphasizing the need for adaptive strategies in dental education during global crises. To address the specific concerns highlighted in this study, future research should delve deeper into the factors influencing student satisfaction, the effectiveness of different online learning modalities, and the long-term implications on clinical skills development. Recommendations for educational institutions include continuous monitoring of student experiences, targeted interventions to address dissatisfaction, and the development of resilient educational models and technologies that can withstand unforeseen disruptions.

## References

[1] Farrokhi F, Mohebbi SZ, Farrokhi F, Khami MR (2021). Impact of COVID-19 on dental education-a scoping review. BMC Med Educ.

[2] Almarghlani AA, Alshehri MA, Alghamdi AA, Sindi MA, Assaggaf MA, Al-Dabbagh NN (2022). Infection-control knowledge, attitude, practice and risk perception of occupational exposure to COVID-19 among dentists: a cross-sectional survey. Niger J Clin Pract.

[3] Linjawi AI, Agou S (2020). E-learning readiness among dental students and faculty members pre-COVID-19 pandemic. J Microsc Ultrastruct.

[4] Alshihri AA, Salem DM, Alnassar TM (2021). A nationwide survey assessing the satisfaction of dental colleges graduates with their undergraduate experience in Saudi Arabia. J Dent.

[5] Alkadi L (2021). Dental education in the COVID-19 era: challenges, solutions and opportunities. Open Dent J.

[6] Mohsin SF, Shah SA, Agwan MA, Ali S, Alsuwaydani ZA, AlSuwaydani SA (2022). Effect of the COVID-19 pandemic on dental interns in Saudi Arabia. Work.

[7] Etajuri EA, Mohd NR, Naimie Z, Ahmad NA (2022). Undergraduate dental students' perspective of online learning and their physical and mental health during COVID-19 pandemic. PLoS One.

[8] Al Rawahi SH, Al Harthy NS, Singh G, Al Isamili MI (2022). Impact of COVID-19 on student's dental education and life. Oman Med J.

[9] Bahanan L, Alsharif M, Samman M (2022). Dental students' perception of integrating E-learning during COVID-19: a cross-sectional study in a Saudi University. Adv Med Educ Pract.

[10] Abdul NS, Alarbash SA, Albati ZH, Alkhelaiwi NK, Alkhalifa WQ, Shenoy M (2022). Impact of covid-19 on education, psychological wellness and life style of dental students in Saudi Arabia. Bioinformation.

[11] Hakami Z, Vishwanathaiah S, Abuzinadah SH, Alhaddad AJ, Bokhari AM, Marghalani HY, Shahin SY (2021). Effects of COVID-19 lockdown on the mental health of dental students: a longitudinal study. J Dent Educ.

[12] Mulla M (2021). Psychological impact of the COVID-19 pandemic on dental hygiene students in Saudi Arabia: a nation-wide study. J Contemp Dent Pract.

[13] Moothedath M (2022). A learning curve is essential to growth: dental education during coronavirus disease 2019. J Pharm Bioallied Sci.

[14] AlFattani A, AlMeharish A, Nasim M, AlQahtani K, AlMudraa S (2021). Ten public health strategies to control the covid-19 pandemic: the Saudi Experience. IJID Reg.

[15] Khan A, Alsofayan Y, Alahmari A (2021). COVID-19 in Saudi Arabia: the national health response. East Mediterr Health J.

[16] Kumar M, Madi M, Vineetha R, Pentapati KC (2022). Impact of the COVID-19 pandemic on graduating dental interns: the students' perspective. Med Pharm Rep.

[17] Javed MQ, Nawabi S, Srivastava S, Kolarkodi SH, Khan AM, Awinashe MV (2023). Undergraduate students' and interns' perception towards learning environment at dental clinics, Qassim University, Saudi Arabia. J Pharm Bioallied Sci.

